# High-Pressure Processing for the Production of Added-Value Claw Meat from Edible Crab (*Cancer pagurus*)

**DOI:** 10.3390/foods10050955

**Published:** 2021-04-27

**Authors:** Federico Lian, Enrico De Conto, Vincenzo Del Grippo, Sabine M. Harrison, John Fagan, James G. Lyng, Nigel P. Brunton

**Affiliations:** 1 UCD School of Agriculture and Food Science, University College Dublin, Belfield, D04 V1W8 Dublin, Ireland; vincenzo.delgrippo@ucd.ie (V.D.G.); sabine.harrison@ucd.ie (S.M.H.); james.lyng@ucd.ie (J.G.L.); nigel.brunton@ucd.ie (N.P.B.); 2 Nofima AS, Muninbakken 9-13, Breivika, P.O. Box 6122, NO-9291 Tromsø, Norway; 3 Department of Agricultural, Food, Environmental and Animal Sciences, University of Udine, I-33100 Udine, Italy; enrico.deco@gmail.com; 4 Irish Sea Fisheries Board (Bord Iascaigh Mhara, BIM), Dún Laoghaire, A96 E5A0 Co. Dublin, Ireland; john.fagan@bim.ie

**Keywords:** HPP, edible crab meat, brown crab, claw muscle, quality attributes, low-field relaxometry, fatty acid profile, microbial inactivation, salt diffusion

## Abstract

High-pressure processing (HPP) in a large-scale industrial unit was explored as a means for producing added-value claw meat products from edible crab (*Cancer pagurus*). Quality attributes were comparatively evaluated on the meat extracted from pressurized (300 MPa/2 min, 300 MPa/4 min, 500 MPa/2 min) or cooked (92 °C/15 min) chelipeds (i.e., the limb bearing the claw), before and after a thermal in-pack pasteurization (*F*_90_^10^ = 10). Satisfactory meat detachment from the shell was achieved due to HPP-induced cold protein denaturation. Compared to cooked or cooked–pasteurized counterparts, pressurized claws showed significantly higher yield (*p* < 0.05), which was possibly related to higher intra-myofibrillar water as evidenced by relaxometry data, together with lower volatile nitrogen levels. The polyunsaturated fatty acids content was unaffected, whereas the inactivation of total viable psychrotrophic and mesophilic bacteria increased with treatment pressure and time (1.1–1.9 log_10_ CFU g^−1^). Notably, pressurization at 300 MPa for 4 min resulted in meat with no discolorations and, after pasteurization, with high color similarity (ΔE* = 1.2–1.9) to conventionally thermally processed samples. Following further investigations into eating quality and microbiological stability, these HPP conditions could be exploited for producing uncooked ready-to-heat or pasteurized ready-to-eat claw meat products from edible crab.

## 1. Introduction

The edible crab (*Cancer pagurus*) is a crustacean species of significant commercial value, which is mainly distributed along the coasts of Western Europe [1]. It is reported that the annual landings of edible crab in Europe have been steadily of about 40,000 t in the last decade [2]. The meat of the claws (i.e., the articles of the cheliped richest in meat) of edible crab is widely appreciated not only for its organoleptic and nutritional properties but also because of its lower cadmium content in comparison with the meat present under the carapace [3]. In line with consumer preference for convenient seafood products, edible crab claws are being increasingly processed into added-value ready-to-eat (RTE) forms, consisting of either picked meat or shell-on products, such as whole or partially de-shelled claws [4].

Generally, industrial crab processing involves a cooking treatment with steam or by immersion in hot water, which may be followed by thermal in-pack pasteurization. The second heat treatment extends shelf life and ensures product safety during prolonged chilled storage [5], whilst the cooking step primarily denatures muscle proteins to achieve detachment of the meat from the exoskeleton, facilitating meat-picking operations [6]. However, thermal processing may have detrimental effects on product yield and quality attributes [7].

An alternative to the use of heat might be the application of high-pressure processing (HPP), whereby products are subjected to elevated pressures (up to 900 MPa) with negligible or minimal thermal exposure [8]. HPP can be applied on mollusks and crustaceans to promote the rupture or loosening of the attachment between the muscle and the calcareous layer of the shell due to cold denaturation of muscle proteins, thus easing the shucking, peeling, and picking operations [9]. Another advantage of HPP is its ability to retain fresh-like nutritional and sensory quality attributes while also achieving enzymatic and microbial inactivation, thus stabilizing the seafood product [10,11].

The application of HPP for facilitating meat picking has been reported to be successful on blue crab (*Callinectes sapidus*) [12]. It has also been explored on Chinese mitten crab (*Eriocheir sinensis*) [13,14], fresh raw edible crab [15], fresh raw mud crab (*Scylla serrata*) [16], and frozen-thawed raw red king crab (*Paralithodes camtschaticus*) [17]. However, to the best of our knowledge, all HPP studies on crab species to date have been conducted only at laboratory- and pilot-scale level, that is, in HPP units with limited capacity (vessel volume 2–55 L) and without the presence of an automatized system for loading the product into the vessel. Furthermore, there has been a lack of in-depth investigations of the knock-on effect of HPP on the quality of claw meat, especially in relation to myowater dynamics and specific chemical constituents of nutritional importance (e.g., fatty acid profile).

Therefore, this study aimed to investigate the effect of different pressure/time treatment combinations applied in an industrial large-scale HPP unit on selected physicochemical, nutritional, and microbiological quality attributes of meat extracted from edible crab claws. Furthermore, in the context of exploring possible commercial implementations for the production of added-value claw meat from edible crab, the quality parameters were also evaluated on the meat of claws that were thermally pasteurized after HPP to assess HPP as a means of producing uncooked ready-to-heat or pasteurized RTE claw meat products. Furthermore, we explored the potential of using HPP for enhancing the diffusion of small solutes (i.e., sodium chloride) into the meat of the claw.

## 2. Materials and Methods

### 2.1. Raw Material

This study was carried out on adult male edible crabs (*Cancer pagurus*), with an average weight of 765 g (±192), purchased from a shellfish processor (Rockabill Seafood, Ltd., Balbriggan, Co. Dublin, Ireland) in September 2017 within one day of landing along the northwest coast of the Irish Sea. The crabs were held at University College Dublin (UCD, Belfield, Dublin, Ireland) at 4 °C in dry live storage before processing within two days of purchasing. During the live storage, the claws of the crabs were immobilized with rubber bands to avoid mutilation or cannibalism phenomena.

On the day of processing, the vitality of the crabs was evaluated, and exemplars that had perished or were showing low vitality were withdrawn from the study. Before processing, the crabs (*N* = 80) were euthanized by piercing the main ganglia. Then, the chelipeds, which are the limbs carrying the claws and constituted the experimental units of the study, were tagged with numbered plastic strips and snapped from the crab body. The chelipeds from different individuals were evenly distributed across different processing treatment groups on the basis of their wet weight.

### 2.2. Processing

#### 2.2.1. High-Pressure Treatments

High-pressure treatments were carried out in an industrial high hydrostatic pressure unit (vessel volume 420 L, model Hiperbaric 420, Hiperbaric, Burgos, Spain) located at an HPP toll processing facility (HPP Tolling Ltd., St. Margaret’s, Co. Dublin, Ireland).

For HPP treatments, the chelipeds were placed into canisters (volume 25 L) filled with fresh water (14 °C) containing 1% (*w*/*v*) sea salt (99.9% sodium chloride, NaCl; British Salt Ltd., Northwich, UK), which functioned as the pressure-transmitting fluid in direct contact with the claws. This sea salt concentration was chosen as it was in line with the salt content (percentage wet weight) of the raw claw muscle (1.10 ± 0.09). In addition, another group of chelipeds was allocated into canisters of the same capacity but filled with fresh water containing 5% (*w*/*v*) sea salt. This allowed for an evaluation of the effect of HPP on salt diffusion into the claw meat.

An equal number of chelipeds (*n* = 30 for each HPP treatment) was pressurized at three different pressure/time conditions, namely at 300 MPa for 2 min (HPP300/2), 300 MPa for 4 min (HPP300/4), or 500 MPa for 2 min (HPP500/2), while immersed in the pressure-transmitting fluid with 1% (*w*/*v*) sea salt. A different group of chelipeds (*n* = 9 for each HPP treatment) was pressurized at the same combinations of pressure/time treatment conditions but while immersed in the pressure-transmitting fluid with 5% (*w*/*v*) sea salt. These pressure/time conditions were chosen based on the results of preliminary trials and previous HPP studies on crab species found in the literature [12,15].

The pressure-transmitting medium contained in the main HPP vessel (i.e., outside the canisters containing the chelipeds) was fresh water at 11 °C. The time to reach the target pressure was 2.0 and 3.5 min for 300 and 500 MPa treatments, respectively, while the decompression was immediate. The temperature of the pressure-transmitting fluids in the canisters and in the HPP vessel increased by less than 1 °C during the HPP treatments. The processed chelipeds were immediately taken to UCD and kept at 4 °C awaiting analytical determinations or further processing.

#### 2.2.2. Water Immersion Cooking

As a comparator to high-pressure processed samples, a group of chelipeds (*n* = 24) was cooked in a thermostatic bath (volume 26 L, model Aqua26Plus, Grant Instruments Ltd., Shepreth, Cambridge, UK) at 92 °C for 15 min by immersion in fresh water containing 1% (*w*/*v*) sea salt. A group of chelipeds (*n* = 3) was cooked under the same conditions but by immersion in fresh water containing 5% (*w*/*v*) sea salt to evaluate the effect of water immersion cooking on salt diffusion into the claw meat as compared with the effect of HPP.

After cooking, the chelipeds were immediately cooled in ice water with 1% (*w*/*v*) sea salt until reaching 20 °C and subsequently kept at 4 °C until analytical determinations or further processing.

Preliminary trials were carried out to determine the cooking and cooling conditions on the basis of time–temperature profiles measured at the core of the cheliped *propodus* (i.e., the largest article of the claw or chela) using K-type thermocouples connected to a data logger (model SQ2040, Grant Instruments Ltd., Shepreth, Cambridge, UK).

#### 2.2.3. Thermal Pasteurization

After HPP or cooking treatments, equal aliquots (*n* = 12) of HPP300/2, HPP300/4, HPP500/2, and cooked chelipeds were further processed by thermal pasteurization. In more detail, each cheliped was vacuum-packed (model C10H, Webomatic^®^ Maschinenfabrik GmbH, Bochum, Germany) individually in boilable 180 μm polyamide/polyethylene (PA/PE) bags (allfo GmbH & Co.KG, Waltenhofen, Germany) and then pasteurized in a thermostatic water bath (model Aqua26Plus, Grant Instruments Ltd., Shepreth, Cambridge, UK) at 95 °C for 25 min. The pasteurization treatment was immediately followed by cooling in fresh water with ice and subsequent storage at 4 °C until analytical determinations. These chelipeds were coded HPP300/2+P, HPP300/4+P, HPP500/2+P, and cooked+P, respectively.

Pasteurization conditions were calculated to deliver an equivalent cumulative lethality value (*F*) of at least 10 min calculated at 90 °C (*z*-value = 10 °C). This *F*-value is the recommended standard to achieve a 6 log inactivation of non-proteolytic *Clostridium botulinum* in RTE low-acid chilled seafood products [18,19]. This pasteurization treatment was conducted to evaluate the effect of a thermal processing step applied after HPP, simulating a possible protocol for the production of RTE meat products from edible crab chelipeds pre-treated with HPP. Moreover, the thermal pasteurization could also account for the effects of an eventual heat treatment in case HPP is used as a pre-treatment for the production of uncooked ready-to-heat crab claw meat products.

### 2.3. Analytical Determinations

All analytical determinations were performed on the muscle meat manually extracted from raw (i.e., untreated) and processed chelipeds (i.e., following HPP, cooking, and thermal pasteurization treatments) using stainless-steel crab picks. All measurements were conducted on the *propodus* (i.e., the part of the claw also constituting the article of the cheliped richest in meat) except colorimetry for which the meat extracted from the *merus* (i.e., the most proximal cheliped article) was used. Thereby, hereinafter, the terms cheliped and claw may be used interchangeably for the purpose of presenting and discussing the results of the present study.

The pH, colorimetry, relaxometry, and calorimetry measurements were completed, respectively, within 4, 8, 24, and 48 h of the processing treatments. The chelipeds used for the analytical determinations of moisture, protein, ash content, total volatile basic nitrogen, fatty acid profile, and salt content were vacuum-packed in PA/PE bags and stored at −80 °C within 24 h of the processing treatments until analyses.

#### 2.3.1. Thermal Transition Properties

The thermal transition properties of crab meat were measured using a differential scanning calorimeter (model DSC 2010, TA Instruments Inc., New Castle, DE, USA) calibrated with indium (melting point 156.6 °C). Differential scanning calorimetry (DSC) was conducted on claw muscle samples homogenized at 8000 rpm for 10 s using an Ultra-turrax^®^ (model DI25, IKA Werke GmbH & Co.KG, Staufen in Breisgau, Germany) to a consistent paste, which was centrifuged at 4000 rpm for 15 min to decrease the moisture content and obtain an enhanced signal. The samples (15–20 mg) were weighed into aluminum pans (TA Instruments Inc., New Castle, DE, USA) and sealed hermetically. An empty hermetically sealed pan was used as a reference. After equilibration at 25 °C, DSC measurements were performed at a heating rate of 7 °C min^−1^ over the range of 25–90 °C under a dry nitrogen flow of 60 mL min^−1^.

The peak temperature (*T*_max_) of protein denaturation and the corresponding denaturation enthalpy (Δ*H*), defined as the area under the denaturation peak, were determined from the generated thermograms using the software OriginPro (version 9.0, OriginLab Corp., Northampton, MA, USA).

#### 2.3.2. Processing Yield

The processing yield was calculated to account for the impact of the processing treatments on cheliped weight as:

Processing yield (%) = (W_processed_ − W_raw_)/W_raw_ × 100 (1)

where W_processed_ is the weight (g) of a processed cheliped, and W_raw_ is the weight (g) of the same cheliped after snapping from the crab prior to any processing treatment.

#### 2.3.3. Moisture, Protein, and Ash Content

The moisture, protein, and ash content of claw meat were determined according to the AOAC methods 950.46, 981.10, and 938.08, respectively [20]. Briefly, the moisture content was quantified by oven drying at 103 °C for 24 h, the ash content was determined after combustion at 550 °C for 16 h, and the protein content was measured by the Kjeldahl method using a conversion factor of 6.25. Results were expressed as percentage of wet sample weight.

#### 2.3.4. Transverse Relaxation Time (T_2_)

Proton transverse relaxation time (*T*_2_) spectra were acquired by low field nuclear magnetic resonance (LF-NMR) to investigate the water distribution and mobility in the samples in line with the method described by Li et al. [21]. Each measurement was performed by running the Carr–Purcell–Meiboom–Gill pulse sequence implemented in an NMR spectrometer (Maran Ultra, Oxford Instruments Ltd., Abingdon, UK) operating at a magnetic field of 0.5 T and a proton resonance frequency of 23.4 MHz. Samples of claw meat (approximately 3 g) were placed into NMR tubes (18 mm outer diameter) and tempered in a water bath (model GD100, Grant Instruments Ltd., Shepreth, Cambridge, UK) at 25 °C for one hour before LF-NMR analysis. The signals were recorded by the RINMR software (version 5.2.0.1, Oxford Instruments Ltd., Abingdon, UK), and each acquisition included 8192 echoes over 32 scans with an inter-pulse spacing (τ) of 150 μs, a relaxation delay of 5 s, and a receiver gain of 1.40. Continuous distributed exponential fitting was applied to phase rotated *T*_2_ data to obtain *T*_2_ continuous distribution curves with the software WinDXP (version 3.0, Oxford Instruments Ltd., Abingdon, UK). The amplitude of the signal was normalized over the unitary area. The area of each peak of the *T*_2_ continuous distribution curves was integrated using the software OriginPro and expressed as proportion (%) relative to the total area under the *T*_2_ curve.

#### 2.3.5. pH

The pH of crab meat was determined in a mixture (1:1) of meat and potassium chloride (KCl; Sigma-Aldrich, Darmstadt, Germany) solution (0.15 M) at room temperature [22]. The mixture was homogenized at 8000 rpm for 30 s using an Ultra-turrax^®^ before analysis with a pH-meter (model HI120, Hanna Instruments Ltd., Leighton Buzzard, UK).

#### 2.3.6. Total Volatile Basic Nitrogen

The total volatile basic nitrogen (TVB-N) was measured according to the method described by Malle and Poumeyrol [23] using a steam distillation unit (model 1002, Foss Analytical A/S, Hillerød, Denmark). Each sample replicate consisted of approximately 5 g of crab meat. Results were expressed as mg nitrogen/100 g of wet sample weight.

#### 2.3.7. Visual Appearance and Color

Images of raw and processed claws were acquired using a digital camera (model DMC-TZ5, Panasonic Corp., Osaka, Japan). The claws were placed on a black felt sheet at a distance of 25 cm from the camera, and the light was provided by two fluorescent tubes. The images were saved in the *jpeg* file format with a resolution of 3456 × 2592 pixels.

The color of the meat extracted from the *merus* was quantitatively determined using a tristimulus colorimeter (model CR-400, Minolta Ltd., Osaka, Japan) with a D65 illuminant and calibrated against a white tile before measurements. The color was expressed in the CIELAB scale as lightness (L*) and green–red (a*) and blue–yellow (b*) coordinates. In addition, to determine the color differences between the HPP samples and the cooked (ΔE*_cooked_) or the cooked-pasteurized (ΔE*_cooked+P_) samples, the parameter ΔE* was calculated as:

ΔE* = [(ΔL*)^2^ + (Δa*)^2^ + (Δb*)^2^]^1/2^. (2)

For each sample, six measurements were taken at different spots on the meat layered onto a Petri dish.

#### 2.3.8. Fatty Acid Profile

The fatty acid profile of crab meat samples was determined by gas chromatographic analysis of fatty acid methyl esters (FAMEs) obtained by microwave-assisted derivatization in a microwave reaction system (MRS, model MARS 6™, CEM Corp., Matthews, NC, USA) according to the method described by Brunton et al. [24].

Each meat sample was analyzed at least in duplicate, and an aliquot of approximately 3 g of meat was used for each analytical replicate.

Reagents of analytical grade (Sigma-Aldrich) were used for the preparation of FAMEs, which started with a saponification step carried out by heating, in the MRS, perfluoroalkoxy reaction vessels (capacity 55 mL) containing the meat sample, 10 mL of 2.5% (*w*/*v*) potassium hydroxide in methanol, and 0.1 mL of internal standard (IS) solution (10 mg/mL tricosanoic acid in chloroform) to 130 °C during 4 min with a holding time of 4 min. After cooling in ice for 5 min, methyl esterification was carried out by adding into the reaction vessels 15 mL of a solution 5% (*v*/*v*) acetyl chloride in methanol and by subsequently heating them in the MRS to 120 °C during 4 min with a holding time of 2 min. After cooling in ice for 5 min, FAMEs were extracted by adding 10 mL of pentane and 20 mL of saturated aqueous sodium chloride (NaCl) solution to the reaction vessels. To facilitate FAME extraction, the reaction vessels were upended both after the addition of pentane and of the saturated NaCl solution. After phase separation, the top layer (i.e., pentane) was aliquoted into 1.5 mL vials containing 0.2 g anhydrous sodium sulfate for analysis using a gas chromatograph (GC) (model Clarus 580, PerkinElmer Inc., Waltham, MA, USA) fitted with a CP-Sil 88 capillary column (100 m × 0.25 mm, 0.2 µm film thickness) (Agilent Technologies Inc., Santa Clara, CA, USA) and a flame ionization detector (FID). The GC-FID operating conditions for separation and quantification of FAMEs were set as reported by Gangopadhyay et al. [25].

The FAMEs were identified by comparing their retention times with analytical standards (Supelco^®^ 37 Component FAME Mix, Sigma-Aldrich, Darmstadt, Germany). The quantification of each fatty acid was based on an internal standard method, using the software TotalChrom (version 6.3.2, PerkinElmer Inc., Waltham, MA, USA) for peak area integration. The fatty acid content was calculated as:

Fatty acid (mg/g sample) = A_FAME_/A_IS_ × W_IS_/W_sample_ × 10 × purity_IS_ (3)

where A_FAME_ and A_IS_ are the peak areas of each FAME and of the IS, W_IS_ and W_sample_ are the weight of the IS (g) and of the sample (g), and purity_IS_ is the purity of the IS.

#### 2.3.9. Microbial Inactivation and Microbial Counts

The effect of HPP on the microbial inactivation was evaluated on the claw meat obtained from crabs euthanized as described in Section 2.1 on the day of purchasing and subsequently stored at 4 °C for two days to allow for the growth of the indigenous microflora prior to the HPP treatments. Raw (i.e., untreated) (*n* = 9) and high-pressure processed (*n* = 3 per HPP treatment) claws were sampled within 12 h of the pressure treatment.

Microbial analyses were also performed on the meat extracted from claws (*n* = 3 per HPP treatment) air-packed in PA/PE bags and stored at 4 °C for two days after the pressure treatment. Meat extraction operations were conducted under aseptic conditions in a laminar flow cabinet (model Bio 48, Faster srl, Ferrara, Italy).

The analyses were performed on claw meat samples (10 g) as a 1:5 dilution in maximum recovery diluent (MRD) (Oxoid Ltd., Basingstoke, UK) following blending in a laboratory blender (model Stomacher^®^ 400 circulator, Seward Ltd., Worthing, UK) at 300 rpm for 2 min. Then, appropriate serial decimal dilutions were prepared in MRD and inoculated on pour-plated plate count agar (PCA; Oxoid Ltd., Basingstoke, UK) supplemented with 0.5% (*w*/*v*) sodium chloride (NaCl; Oxoid Ltd., Basingstoke, UK) incubated at 10 °C for 7–9 days for the enumeration of the total viable psychrotrophic counts (TVC_p_) or at 30 °C for 3 days for the enumeration of the total viable mesophilic counts (TVC_m_). Microbial counts were reported as the decimal logarithm of colony-forming units per gram of sample (log_10_ CFU g^−1^). Microbial inactivation was expressed as an absolute value and calculated as log_10_(*N*/*N*_raw_), where *N*_raw_ is the count in raw samples, and *N* is the count in processed samples.

#### 2.3.10. Salt Content

The salt content of crab meat was determined using the method described by Lascorz et al. [26] with some modifications. Briefly, aliquots of 2 g of crab meat were added to 100 mL of an aqueous 0.1 N nitric acid (HNO_3_) solution (Sigma-Aldrich) and homogenized at 8000 rpm for 10 s using an Ultra-turrax^®^. The homogenate was placed into a shaking water bath at 65 °C for 15 min and subsequently cooled on ice to a final temperature of 20 °C before titration against 0.1 N silver nitrate (AgNO_3_; Sigma-Aldrich) using a pH meter equipped with a silver electrode to an endpoint of +220 mV. The salt content was expressed as percentage of wet sample weight using the following formula:

Salt content (%) = (A − B) × 0.585/C(4)

where A is the volume (mL) of AgNO_3_ titrating the sample, B is the volume (mL) of AgNO_3_ titrating the blank, and C is the sample weight (g).

### 2.4. Statistical Analysis

Data analysis was performed considering each claw generated from different crabs as an independent biological replicate. The results were expressed as mean values (±standard deviation) of at least three different biological sample replicates per treatment. Differences between treatments were evaluated by one-way analysis of variance (ANOVA) followed by post-hoc multiple comparisons (Tukey’s HSD test). For salt content data, a one-way ANOVA followed by post-hoc Dunnett’s test was performed to assess significant differences from the values obtained for the meat samples of raw (i.e., untreated) claws. The statistical tests were carried out at a 5% probability level (*p*-value) using the software Statistica™ (version 8.0, StatSoft Inc., Tulsa, OK, USA).

## 3. Results and Discussion

### 3.1. Thermal Transition Properties of Claw Muscle Proteins

**Figure 1:**
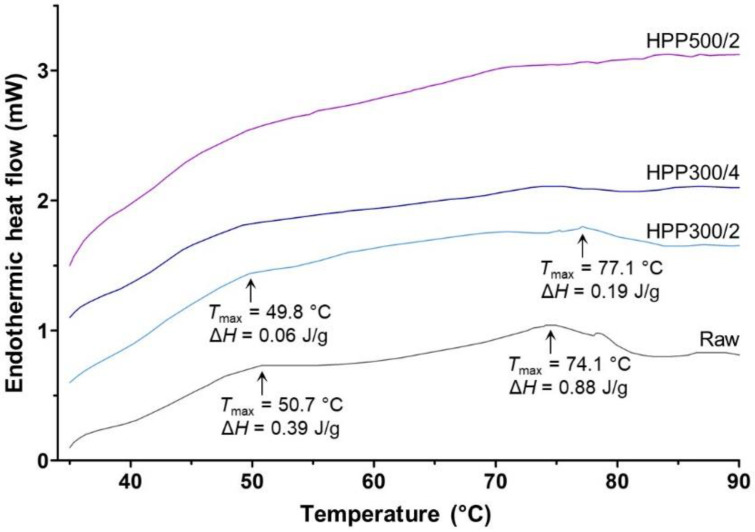
Thermograms obtained from differential scanning calorimetry of meat extracted from raw and high-pressure processed edible crab claws. Detected thermal transition peaks are indicated with arrows accompanied by corresponding protein denaturation temperatures (*T*_max_) and enthalpy (Δ*H*).

The effect of HPP on the thermal transition properties of the meat extracted from edible crab claws is illustrated by the thermograms in Figure 1.

Two main thermal transition peaks were detected for raw meat with maximum temperatures (*T*_max_) of 50.7 and 74.1 °C. These peaks could be ascribed, respectively, to the denaturation of myosin and actin, which is in agreement with the data reported for claw muscle of mud crab (*T*_max_(myosin) = 45–47.5 °C, *T*_max_(actin) = 72.4 °C) [27], blue crab (*T*_max_(myosin) = 48.2 °C, *T*_max_(actin) = 76.8 °C) [12], and Southern Ocean swimming (*Ovalipes trimaculatus*) and Patagonian stone (*Platyxanthus patagonicus*) (*T*_max_(myosin) = 49.0 °C, *T*_max_(actin) = 77.5 °C) crabs [28]. No direct comparison with thermograms acquired for claw muscle of edible crab could be found in the literature.

As an effect of HPP, decreasing residual protein denaturation enthalpy (Δ*H*) was observed in the pressurized meat samples with increasing treatment pressure and time. The myosin and actin endothermic peaks of the samples treated at 300 MPa were poorly defined, while no peaks were detected for HPP500/2 samples. A similar trend was reported for the claw meat of blue crab pressurized in the range of 100–600 MPa for 5 min, where the authors attributed the observed denaturation of myosin and actin to modifications of non-covalent protein interactions (e.g., electrostatic and hydrophobic) and the subsequent reformation of intra- and inter-molecular bonds within or between protein molecules [12]. Furthermore, this is in line with the often-cited contention that treatments at pressures higher than 150–200 MPa lead to tertiary and quaternary protein structure changes [29].

**Figure 2:**
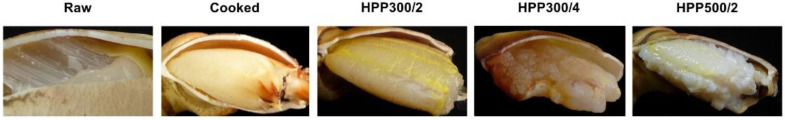
Detail of the detachment of the muscle tissue from the inner layer of the cuticle in cooked and high-pressure processed chelipeds of edible crab (*Cancer pagurus*) compared with their raw (i.e., untreated) counterparts. Sample codes: HPP300/2 and HPP300/4, high-pressure processed at 300 MPa for 2 and 4 min, respectively; HPP500/2, high-pressure processed at 500 MPa for 2 min.

In crustaceans, claw and leg muscles are anchored to the exoskeleton through extensive interdigitated junctions in which, among muscle fibers, only thickened I-bands of actin are involved in the connection on the muscle side [30]. This supports the hypothesis that the protein denaturation induced by HPP can break the attachment between shell and muscle [31], provided that the pressure conditions allow for sufficient denaturation of actin. All the HPP treatments applied in this study successfully allowed for complete detachment of the muscle tissue from the inner layer of the cuticle (Figure 2), as also evidenced by the resulting facilitated meat picking. By contrast, preliminary trials conducted at 250 MPa for 2 min (data not shown) were found not to deliver sufficient detachment of the muscle from the shell to ease picking. This is also in accordance with research on blue crab pressurized below 300 MPa, where the total extractable meat was not significantly higher than in raw counterparts [12].

### 3.2. Processing Yield and Moisture, Protein, and Ash Content

The processing yield and the moisture, protein, and ash content of raw and processed claw meat samples are reported in Table 1.

The processing yield was significantly lower (*p* < 0.001) in cooked claws compared to the pressurized claws, in which a slight weight gain was observed as a result of HPP, similarly to the results reported for red king crab pressurized at 260 MPa for 1.5 min [17]. The observed cook loss (5.2% ± 1.2) is comparable to that reported in a study on the cooking yield of whole edible crab [32] but lower than that reported for claw meat of boiled edible crab (5–15%) [33]. This is likely because of the fact that in our study, a comparatively milder cooking treatment was employed, which was specifically designed to reflect an industrial set-up for the processing of snapped claws rather than for whole crabs. On the other hand, the slight weight gain observed in the pressurized samples could be explained by the ingress of water into the muscle tissue as a consequence of the direct contact of the claws with the pressure-transmitting fluid (i.e., fresh water with 1% (*w*/*v*) sea salt) and the presence of a dense network of pore canals (i.e., 150,000–220,000 mm^−2^) in the exoskeleton of edible crab [34]. Notably, limited weight loss occurred during the subsequent thermal pasteurization of pressurized claws, and the final processing yield for HPP+P claws was higher than for both cooked and cooked+P claws, respectively, by 3.8–4.6% and 6.3–7.1% calculated on a raw claw weight basis. These results indicate that, for edible crab processors, HPP may be more profitable than conventional cooking for the production of claw meat products.

The moisture, protein, and ash content of raw meat was comparable to that reported for claw muscle tissue of male edible crabs caught in geographic areas close to the Irish Sea [35]. The moisture content in cooked meat samples was lower than in their raw and pressurized counterparts by 2.2 and 3.8–7.1 (g/100 g meat wet weight), respectively. This could be ascribed to the conformational changes occurring to proteins in the muscle during the cooking process, including unfolding, aggregation, and gelification with consequent shrinkage of the myofibril lattice into a denser structure, causing the release of water pressed out from the sarcoplasm and myofibrils [36,37]. The observed differences in moisture content indicate that HPP may induce protein conformation changes to a lesser degree than heat [38]. Furthermore, higher moisture levels were found with increasing treatment pressure and time.

Overall, the moisture, protein, and ash content results confirm that the observed differences in processing yield can be mainly ascribed to the higher water content in the muscle tissue, which was likely due to increased protein hydration induced by HPP treatments [39].

Interestingly, after thermal pasteurization, the moisture content remained higher in the samples that had previously been pressurized compared with previously cooked samples, with the highest value observed for the treatment HPP300/4+P. The destabilization of myosin heads, which are particularly pressure-sensitive, may affect the formation of an actomyosin complex, possibly hindering sarcomere shortening and the consequent liquid loss generated by the compression of myofibrils [40]. At least to some extent, these events may promote water retention during pasteurization, as observed for heat-pasteurized cod, which had previously been pressurized at 100 MPa [41].

### 3.3. Muscle Water Distribution and Mobility (Transverse Relaxation Time, T_2_)

Transverse relaxation time (*T*_2_) measurements were carried out to gain further insights regarding the effect of HPP and thermal treatments on myowater dynamics. In muscle tissues, the components of *T*_2_ spectra reflect the interactions between water and proteins, particularly concerning water distribution, compartmentalization, and mobility within the muscle structure. The relaxation time of a given *T*_2_ component is directly related to the mobility degree of the corresponding water population, whereas the peak area relates to the amount of water belonging to a specific relaxation component [42].

**Figure 3:**
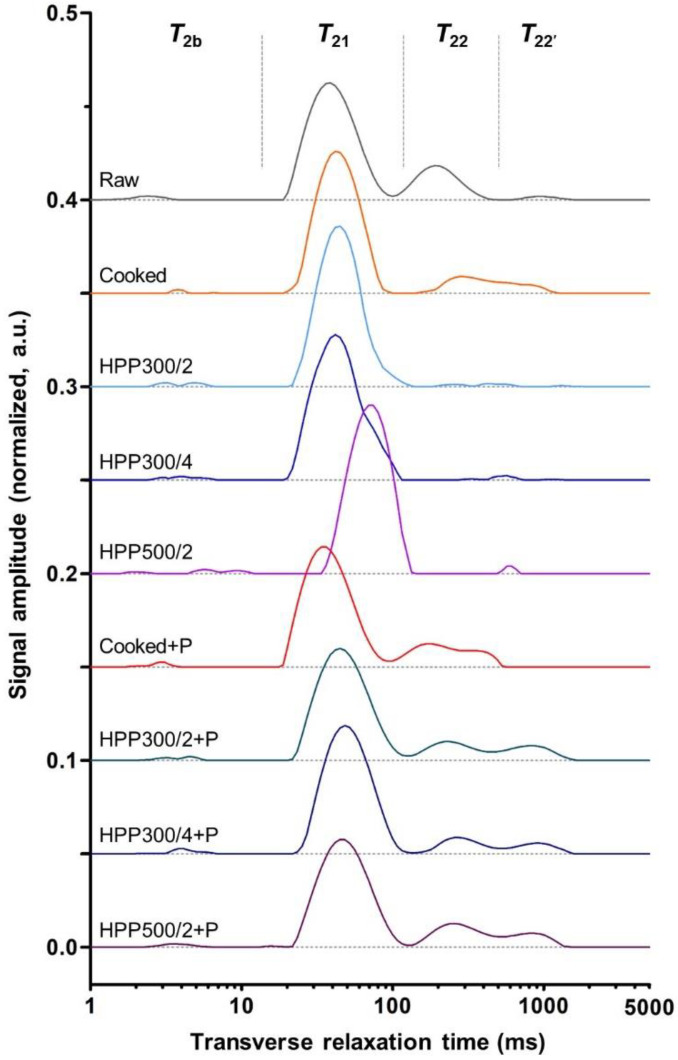
Continuous distribution curves of transverse relaxation time (*T*_2_) obtained by LF-NMR analysis of meat extracted from raw (i.e., untreated), cooked, and high-pressure processed claws, before and after thermal pasteurization (indicated with +P). The signal amplitude is expressed in arbitrary unit (a.u.) and normalized over unitary area. Water relaxation components are indicated with *T*_2b_, *T*_21_, *T*_22_, and *T*_22__′_.

The *T*_2_ continuous distribution curves obtained for the meat of raw and processed claws showed the presence of four peaks (Figure 3), each corresponding to a specific water population as described in LF-NMR studies on shrimp and Chinese mitten crab muscle [21,43,44,45]. More specifically, the peak registered at about 1–10 ms (*T*_2b_) may be ascribed to water tightly bound to macromolecules (e.g., lipids or proteins), the component in the range of 20–120 ms (*T*_21_) may be associated with water entrapped or immobilized in the intra-myofibrillar space between the thick and thin muscle filaments, the peak *T*_22_ (120–500 ms) may represent extra-myofibrillar water loosely held between myofibrils, and the peak *T*_22__′_ (500–1500 ms) may account specifically for the free extra-myofibrillar water located in the space between collagen fibrils in the myofibril lattice.

Table 2 shows that the cooking process increased the relaxation time of intra-myofibrillar water (*T*_21_), while there was no variation observed in the relative proportion of *T*_21_. By contrast, all pressure treatments led to a dramatic increase (from 37.4 to 79.8–90.4%) in the relative *T*_21_ area together with a significant (*p* < 0.05) increase (by about 34 ms) in the mobility of this component in HPP500/2 samples.

Regarding the extra-myofibrillar components, cooking resulted in a substantial redistribution of the extra-myofibrillar water with a significantly (*p* < 0.05) larger population of free extra-myofibrillar water (*T*_22′_) together with a significant (*p* < 0.05) increase (by about 100 ms) in the mobility of the *T*_22_ extra-myofibrillar water pool. Longer *T*_22_ relaxation times were also observed for pressure-treated samples, but in this case, they were accompanied by a decrease in the amount of extra-myofibrillar water (i.e., the total area of *T*_22_ and *T*_22′_), which was composed of a single relaxation component in HPP300/4 and HPP500/2 samples. While our results are in agreement with the study of Shang et al. [46] on sea bass skeletal muscle treated at 100–600 MPa for 10 min, they are in contrast with the assumptions reported in the works of Dang et al. [31], Kaur et al. [47], and Yi et al. [48], where it was hypothesized a relocation of water from the intra- to the extra-myofibrillar space as a direct effect of HPP on black tiger shrimp (*Penaeus monodon*) after treatment at 100–435 MPa for 5 min and on bay scallop (*Argopecten irradians*) treated at 150–400 MPa for 2–3 min.

The dissimilarities in the *T*_2_ spectra between cooked and pressurized samples might be ascribed to differences between heat- and pressure-induced denaturation mechanisms and related changes in the myofibrillar architecture. Heat can destabilize proteins by the rearrangement of non-polar hydrophobic ends from the hydrophobic core toward the water [8], leading to contraction of the myofibrillar network, myosin protein deformation, and an increase of the extracellular space [21,38], as shown by microstructural imaging of shrimp muscle in the work of Niamnuy et al. [37]. This may explain the redistribution of the extra-myofibrillar water to free water, as observed after cooking [45]. Conversely, pressure denaturation is associated with the forcing of water molecules into the inner space of the protein matrix [49]. The infiltration of water is also favored by other pressure-induced changes, such as the collapse of hydrophobic cavities and the promotion of protein–water hydrogen bonds over electrostatic and hydrophobic interactions, which may increase the hydration capacity of proteins [50]. Consequently, the infiltrated water causes swelling of the hydrophobic protein core [51], which can explain not only the increase in the peak area but also the longer relaxation time of the *T*_21_ component, especially at high pressure levels (HPP500/2). In fact, Bertram et al. [52] have related the *T*_21_ relaxation time to the distance between the thick and thin muscle filaments, and in this way, it can be considered an indicator of myofibrillar swelling [53]. Likewise, the single extra-myofibrillar component observed for HPP300/4 and HPP500/2 samples can be explained by the compaction of the muscle tissue with consequent rearrangement of the extracellular inter-myofibrillar space, as evidenced by scanning electron microscopy analysis in black tiger shrimp pressurized at 100, 270, and 435 MPa for 5 min [47] and razor clam (*Sinonovacula constricta*) treated at 200, 300, and 400 MPa for 3 or 10 min [54].

Overall, the presented relaxation data support the hypothesis that the higher processing yield and moisture content observed for HPP samples can be mainly ascribed to the ingress of the pressure-transmitting water medium into the intra-myofibrillar network.

After the pasteurization of cooked samples, a general broadening of the peaks was observed together with a significant (*p* < 0.05) reduction of the mobility of both extra-myofibrillar components along with a significant (*p* < 0.05) decrease (by about 7 ms) in *T*_21_ relaxation time. This might be explained by the fact that the double heat treatment causes lateral shrinkage of myofibrils as well as a loss of compartmentalization between intra- and extra-myofibrillar spaces [55].

By contrast, the pasteurization of pressurized samples did not affect the *T*_21_ relaxation time but drastically decreased the *T*_21_ peak areas (from 79.8–90.4 to 26.1–37.0%) with water migration into the *T*_22__′_ component. The highest total extra-myofibrillar water was observed in HPP500/2+P samples. This may indicate that the HPP-induced infiltration of water into the intra-myofibrillar space observed in the present study was likely associated with unstable protein–water interactions due to damaged tissue structures [56]. In fact, it has been shown that HPP can alter the original myofibrillar architecture with the formation of voids between muscle fibers, especially at high pressures, resulting in a release of sarcoplasmic proteins from interstitial spaces and the formation of holes between fibers [47]. The application of thermal pasteurization after pressure treatment can cause additional damage to the muscle tissue structure with further destabilization of protein–water interactions, hence triggering the redistribution of the water, which was previously pushed by HPP into the interior of the protein matrix, from the intra- to the extra-myofibrillar space [48].

An increase in the peak areas and relaxation time of extra-myofibrillar water populations (*T*_22_ and *T*_22__′_) has previously been correlated to a decrease in the water-holding capacity (WHC) [21,55] and potential drip or purge loss [57]. In this regard, according to the relaxation data obtained in the present study, pressurized meat might be characterized by higher WHC compared to cooked meat, as shown by Martínez et al. [12]. At the same time, poorer WHC properties might be assumed for the pressurized samples after the pasteurization treatment, given the substantial water mobilization toward the most mobile component with a long relaxation time.

Nevertheless, it is noteworthy that high water mobility should not be necessarily considered an indicator of low eating quality. As an example, juiciness, which is an important sensory driver of liking in RTE crab claw meat [58], has been shown to be related to a high amount of mobile water in meat products [59]. Supporting this hypothesis, blue crab meat after HPP and subsequent thermal pasteurization was found to be significantly juicier than its conventionally cooked counterpart [12].

### 3.4. pH and Total Volatile Basic Nitrogen

The pH and total volatile basic nitrogen (TVB-N) values of raw and treated claw meat samples are reported in Table 1.

The pH of raw meat was in line with the values observed for other crab species, such as crucifix crab (*Charybdis feriatus*) [60] and snow crab (*Chionoecetes opilio*) [22]. The pH of crab meat is inherently higher than other seafood species due to the higher content in non-protein nitrogenous (NPN) compounds (e.g., trimethylamine oxide, peptides, and free amino acids) [61].

As an effect of cooking, the pH increased by approximately 0.4 of a unit to pH values similar to the ones reported for claw meat (pH 7.4) of freshly cooked edible crab [62], which was possibly due to the depletion of protons in the muscle [22] as well as due to the presence of basic ammonia-like compounds formed from the thermal decomposition of tissue proteins and NPN compounds [62].

HPP resulted in higher pH values than raw meat with increasing treatment pressure and time. Similarly, a slight increase in pH values was reported by other authors for HPP-treated black tiger shrimp [47] and Indian white prawn (*Fenneropenaeus indicus*) pressurized for 5 min at 100, 270, 435, and 600 MPa [63]. The increase in pH induced by HPP might be connected with the denaturation-related changes in the tertiary and quaternary protein structure, which may cause the exposure of alkaline amino acids [40], such as arginine and lysine that are abundant in crab meat [35]. Furthermore, at pressures greater than 300 MPa, the rupture of hydrogen bonds may promote the exposure of hydrophobic sites, leading to a reduction in proton mobility [40].

After pasteurization, the pH increased significantly (*p* < 0.05) in all samples. However, in HPP300/2+P and HPP300/4+P samples, the pH remained significantly (*p* < 0.05) lower as compared with cooked and cooked+P samples. This might be explained by a buffering effect of proteins, deriving from the conformational changes caused by the previous treatment at 300 MPa, possibly counteracting the increase in pH linked to thermal degradation during the pasteurization [40].

The TVB-N value (i.e., level of amine and ammonia compounds) for raw meat was comparable to the literature data for other crab species, such as crucifix crab [60], snow crab [22], and red snow crab (*Chionoecetes japonicus*) [64]. The cooking process increased the TVB-N level significantly (*p* < 0.001), which was most likely as a result of a thermal breakdown of proteins and NPN compounds [62,65]. By contrast, HPP caused a decrease in TVB-N values with increasing treatment pressure and time. A similar trend was observed in HPP-treated Indian white prawns [63], where the TVB-N values decreased from 15.5 to 13.1 and 9.6 mg N/100 g after pressurization for 5 min at 100 and 600 MPa, respectively. This effect might be ascribed to a dilution caused by the ingress of water into the muscle and the solubilization into the pressure-transmitting water medium of part of the TVB-N compounds present.

After pasteurization, TVB-N values in previously pressurized claws were significantly (*p* < 0.05) lower (by 12.2–17.8 mg N/100 g) than in their cooked-pasteurized counterparts. This may also indicate that NPN compounds are, at least in part, drained away from the muscle during HPP, limiting the formation of TVB-N occurring due to their thermal degradation upon pasteurization.

The European legislation sets a TVB-N limit of 25–35 mg N/100 g for fishery products, although it is not inclusive of crustacean species [66]. In the present study, this limit was exceeded only by cooked+P and HPP500/2+P samples. It should be noted that a low initial value of TVB-N in freshly processed crab meat is desirable as TVB-N increases during storage as a result of enzymatic activity exacerbated by microbial growth. Notably, undesirable ammonia-like odors and flavors in crab meat have been related to high TVB-N levels, constituting a possible cause of sensory rejection of the product [22].

### 3.5. Visual Appearance and Color

Overall, visual appearance and color are important quality features in seafood products, playing a key role in consumers’ purchasing choice [67].

**Figure 4:**
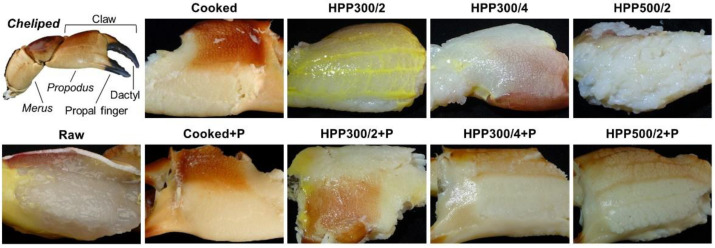
Illustration of the anatomy of a cheliped of edible crab (*Cancer pagurus*) and images of meat of the *propodus* of raw (i.e., untreated), cooked, and high-pressure processed claws, before and after thermal pasteurization (indicated with +P).

An overview of the visual appearance of the *propodus* of differently processed chelipeds is illustrated in Figure 4. The overall appearance of this anatomic part is of particular importance, as it is often commercialized as a standalone product in a partially or totally de-shelled form using clear packaging (e.g., the so-called “cocktail claws”).

A yellow discoloration could be observed in the claws pressurized at 300 MPa for 2 min. Most likely, this was due to the pressure treatment causing the spreading of the yellow tissue, which was possibly associated with the muscle tendon and, therefore, of proteinaceous nature, present in the *propodus* in proximity to the dactyl and the propal finger of the claw [68]. Likewise, a scrambled-egg-like appearance was reported by other authors after treatment of edible crab claws at 250 and 300 MPa for 2.5 min [15]. By contrast, this discoloration was hardly noticeable in HPP300/4 samples, whereas it was not present in HPP500/2 claws, most likely because of pressure-induced denaturation of the yellow tissue.

HPP300/4 samples were characterized by a smooth surface and a distinctive purple-hued area. This may be ascribed to the presence in the muscular epithelium of a blue-brown protein-carotenoid complex, which is partially dissociated upon HPP at 300 MPa [69], causing, to a certain degree, the release of red astaxanthin. Thus, the observed purple color could derive from a mixture between the color of complexed and free astaxanthin [31].

Compared with the claws pressurized at 300 MPa, HPP500/2 meat samples were whiter, brighter, and more opaque, although they were also characterized by a “lumpier” surface appearance, which was likely as a result of the formation of protein aggregates at elevated pressure levels due to the compaction of the gap between muscle fibers [47,54] and the promotion of inter-protein interactions [51]. These aggregates are stabilized by disulfide bonds with the exposure of buried sulfydryl groups [8], possibly explaining the marked sulfuric off-odor, which, although not quantitatively assessed, was distinctively perceived on HPP500/2 claws. This off-odor was still slightly perceivable in HPP500/2 claws after pasteurization. Although this observation warrants further investigation, possibly via analytical examination of volatile odor compounds, it is in agreement with the study of [15], who reported off-odors resembling garlic or rotten eggs in edible crab after HPP. By contrast, no specific undesirable notes were reported for thermally pasteurized claw meat extracted from HPP-treated blue crab [12].

After pasteurization, the appearance of HPP300/4 samples resembled that of cooked or cooked+P samples, as heat caused severe protein denaturation; hence, the color of free red astaxanthin became dominant [31]. Likewise, following pasteurization, the “lumpy” appearance of HPP500/2 samples became smoother and more similar to that of cooked or cooked+P samples. By contrast, the yellow discoloration was still visible on HPP300/2 claws after pasteurization, suggesting that HPP might cause changes in the yellow tissue so that its color is no longer transformed to an orange hue by a subsequent thermal treatment.

The color was quantitatively determined by using a colorimeter on the meat of the *merus* (Table 1).

Lightness (L*) increased with treatment pressure and time, resulting in significantly (*p* < 0.05) higher values in HPP500/2 samples as compared with the claws treated at 300 MPa, also after pasteurization. Most likely, the HPP-induced protein coagulation changed the ratio between absorbed and reflected light at the meat surface, leading to a whiter color appearance [70,71], as also shown in American lobster (*Homarus americanus*) tails pressurized at 350 MPa for 5–10 min [72]. Notably, after pasteurization, the lightness of samples pressurized at 300 MPa was not significantly (*p* ≥ 0.05) different from cooked and cooked+P meat. Lightness is an important color attribute in crab meat, as confirmed by previous studies reporting higher sensory scores for whiter blue crab meat [12] and associating a vivid appearance (i.e., color attributes such as white, bright, glossy, or shiny) of RTE edible crab claw meat to higher consumer liking [58].

A significant (*p* < 0.05) decrease in redness (a*) was observed with increasing treatment pressure, and, after pasteurization, the redness increased in all pressurized samples, reaching values comparable to that of cooked and cooked+P samples. A similar trend was observed for yellowness (b*).

In general, considering the color difference values (ΔE*), the color of pressurized meat was distant from that of cooked (ΔE*_cooked_ = 8.2–11.8) and cooked+P (ΔE*_cooked+P_ = 8.6–12.2) samples. However, after the application of a thermal process (i.e., pasteurization), the meat color became more similar to that of cooked and cooked+P samples. In particular, the ΔE*cooked and ΔE*_cooked+P_ values of HPP300/2+P and HPP300/4+P samples were in the range of 1.2–2.0, meaning that the color differences were only slightly distinguishable by the human eye [40]. This may constitute a promising quality aspect for consumer acceptance of HPP-treated ready-to-heat and HPP-treated pasteurized RTE products.

### 3.6. Fatty Acid Profile

The fatty acid composition of raw and processed claw meat is presented in Table 3.

The values and variability observed for raw meat were in line with fatty acid data previously reported for edible crab claw muscle [35]. Specifically, saturated fatty acids (SFAs) were mainly composed of palmitic (16:0) and stearic (18:0) acid; the main monounsaturated fatty acids (MUFAs) included palmitoleic (16:1), oleic (18:1*n*−9*t*), and *cis*-vaccenic (18:1*n*−7) acid; whereas the most abundant polyunsaturated fatty acids (PUFAs) were arachidonic (20:4*n*−6), eicosapentaenoic (EPA, 20:5*n*−3), docosapentaenoic (22:5*n*−3), and docosahexaenoic (DHA, 22:6*n*−3) acid.

Regarding the effect of processing on the fatty acid composition, the level of palmitic acid in cooked–pasteurized claws was significantly (*p* < 0.05) higher than in HPP300/2 samples. Similarly, the level of stearic acid was significantly (*p* < 0.05) higher in cooked claws as compared with HPP300/4 samples. As a result, the highest proportion of SFAs was found in cooked and cooked+P samples, although these values were not significantly different (*p* ≥ 0.05) from raw or HPP-treated samples. Furthermore, while no significant differences (*p* ≥ 0.05) between treatments were observed for the main MUFAs and PUFAs, the total amount of MUFAs in cooked+P samples was significantly (*p* < 0.05) higher than in HPP300/2+P claws. By contrast, the meat of cooked+P claws contained a significantly (*p* < 0.05) lower total amount of PUFAs as compared with HPP300/2+P samples.

Among PUFAs, there is a particular interest in assessing the stability of omega−3 fatty acids, especially EPA and DHA, in light of their purported beneficial anti-inflammatory and immunomodulatory properties [73]. Furthermore, the ratio between omega−3 and omega−6 PUFAs is of nutritional importance as it is a key factor for a balanced synthesis of eicosanoids [74], with values close to 1 or higher recommended for the human diet [75]. In the present study, the ratio omega−3/omega−6 ranged 4.4–6.6 and was not significantly (*p* ≥ 0.05) affected by processing. Moreover, irrespective of the processing treatment, the total amount of EPA and DHA was above the minimum threshold (i.e., 80 mg per 100 g and 100 kcal of product) required within the European Union for bearing the label claim *high in omega-3 fatty acids* [76].

It should be noted that the observed differences in the fatty acid composition, although in some cases statistically significant (*p* < 0.05), were relatively small, with percentage values differing in absolute terms by less than 4% between treatments. Our results are in contrast with the study of Maulvault et al. [33], reporting a marked reduction in PUFAs in claw meat of edible crab after cooking treatments, which was explained by the oxidation and fragmentation phenomena promoted by high temperatures. On the contrary, with regard to the effect of HPP on the stability of the fatty acid composition, our results appear in agreement with the findings of previous works on shellfish reporting a lack of substantial changes after pressure treatments at up to 600 MPa [77,78].

Nonetheless, it has been suggested that HPP may trigger lipid oxidation reactions through the rupture of cellular membranes, resulting in the exposure of unsaturated lipids to oxidation catalysts such as metal ions and enzymes (e.g., lipases or lipoxygenases) [79]. As a result of oxidation, the proportion of PUFAs may decrease concomitantly with an increase in SFAs; however, these effects would become apparent only at advanced oxidation stages [80]. In the present study, the type of processing did not have a significant (*p* ≥ 0.05) impact on the ratio PUFA/SFA or the polyene index (PI), which is calculated as the ratio between the sum of EPA and DHA and the palmitic acid and is considered a valuable measure of oxidation levels [81]. Stable PUFAs levels and no significant changes in the PI value were also observed, respectively, in Atlantic salmon (*Salmo salar*) fillets treated at 150 MPa or 300 MPa for 15 min [82] and in coho salmon (*Oncorhynchus kisutch*) fillets pressurized at 135, 170, or 200 MPa for 30 s [83] during chilled storage. Nonetheless, as future research, it remains of interest to evaluate HPP-treated edible crab meat for the stability of its fatty acid profile during storage.

### 3.7. Microbial Inactivation and Microbial Counts

**Figure 5:**
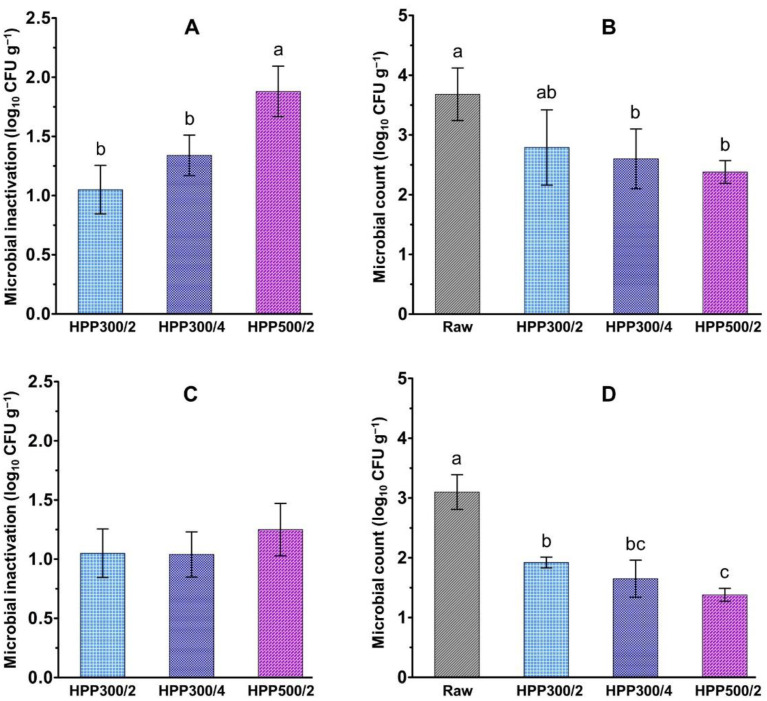
Left side: HPP-induced inactivation (log_10_ CFU g^−1^) of total viable psychrotrophic (**A**) and mesophilic (**C**) counts in claw meat. Right side: Total viable psychrotrophic (**B**) and mesophilic (**D**) counts (log_10_ CFU g^−1^) in the meat extracted from the claw of raw (i.e., untreated) and high-pressure processed chelipeds stored for two days at 4 °C after crab euthanization or HPP treatment. Error bars indicate standard deviations of the mean values. Different letters within a sub-plot indicate significantly (*p* < 0.05) different mean values.

The effect of HPP on microbial inactivation and the microbial counts in raw (i.e., untreated) and pressure-treated samples 48 h after crab euthanization or HPP treatment are illustrated in Figure 5.

All the pressure treatments reduced the total viable psychrotrophic (TVC_p_, Figure 5A) and mesophilic (TVC_m_, Figure 5C) bacteria count. In general, the microbial inactivation increased with treatment pressure and time and was higher for the psychrotrophic than the mesophilic population. In particular, the inactivation of TVC_p_ obtained after treatment at 500 MPa for 2 min (1.9 ± 0.2) was significantly higher (*p* < 0.05) than that achieved by applying the treatment at 300 MPa for 2 min (1.2 ± 0.2) or 4 min (1.3 ± 0.2).

Comparable inactivation results were obtained in previous HPP studies on crab and seafood species in other studies. For example, Leadley et al. [15] reported a reduction by 1.3 log_10_ CFU g^−1^ of the total viable mesophilic count of edible crab meat following HPP treatment at 300 MPa for 2.5 min. Furthermore, pressure treatments at 300 and 550 MPa for 5 min applied to vacuum-packed cooked blue crab meat inactivated the total mesophilic aerobic count by 0.8 and 2.1 log_10_ CFU g^−1^, respectively, starting from a microbial contamination of 5.1 log_10_ CFU g^−1^ [84]. Linton et al. [85] reported that pressure treatments between 300 and 600 MPa for 2 min resulted in a slight reduction of the total mesophilic count in Norway lobster (*Nephrops norvegicus*), while the psychrotrophic count was significantly reduced by 2 log_10_ CFU g^−1^ after pressure treatment at 300 MPa. A pressure treatment at 550 MPa for 5 min decreased by more than 2.25 log_10_ CFU g^−1^ the total viable mesophilic count in RTE wine-marinated Pacific white shrimps (*Litopenaeus vannamei*) [86].

To acquire information on the effect of HPP on microbial quality during post-treatment refrigerated storage, TVC_p_ and TVC_m_ were determined in the claw meat extracted from raw and pressure-treated crabs 48 h after crab euthanization and HPP treatment (Figure 5B,D).

Microbial counts in the raw (i.e., untreated) meat 48 h after euthanization were within the range reported in the literature for edible crabs sampled two days post mortem [87]. Notably, HPP treatments at 300 MPa for 4 min and 500 MPa for 2 min resulted in meat samples with significantly (*p* < 0.05) lower TVC_p_ and TVC_m_ values than in the raw meat. Most likely, the HPP treatments caused damage to the microbial cells [85], hindering their ability to grow for at least the first two days of the subsequent refrigerated storage.

This indicates that HPP may help retain a higher microbial quality in the product for at least the first 48 h of refrigerated storage. This information may be particularly valuable for crab processors that are aiming to exploit HPP as a means of producing added-value crab meat products that would require a certain time before further processing can be applied to the product, for example, to allow for the transport of the pressure-treated product from the HPP facility to the production plant.

On the other hand, it should also be noted that the microbial stability of the pressure-treated product should be carefully evaluated if HPP is not followed by adequate heat processing and the product undergoes prolonged refrigerated storage [88]. If spore-forming bacteria are present, they would require more severe pressure/time conditions than vegetative cells for inactivation [89]. In addition, pressure treatments below 600 MPa may trigger the germination of spore-forming bacteria [90]. In fact, the presence of spore-forming species with pathogenic potential such as *Bacillus weihenstephanensis* has been reported in the meat of edible crab [91]. Non-proteolytic strains of *Clostridium botulinum* spores may also be potentially present in crab meat products, mainly arising from contamination from the gills of the animal during slaughtering and processing operations [92]. This may constitute a possible hazardous scenario as HPP may inactivate vegetative bacteria responsible for the off-odors often associated with seafood spoilage. At the same time, the meat would support the proliferation of spore-forming bacteria, possibly leading to toxin production [93], especially if the pressure treatment is followed by storage in modified atmosphere or vacuum packaging [85]. *Clostridium* spp. was the dominant bacteria during the refrigerated storage of Chinese mitten crab meat shucked by HPP at 300 MPa for 20 min [13].

Therefore, further investigation of the microbial quality during the storage of pressure-treated crab meat products is warranted. However, pending these further studies, crab processors may replace the cooking step with HPP for the commercialization of pressure-treated crab meat products in an RTE form only after the application of adequate heat pasteurization (*F*_90_^10^ = 10). Alternatively, the high-pressure processed product may be commercialized in a frozen state while providing the final consumer with indications that the product should be cooked soon after thawing (i.e., ready-to-heat product).

### 3.8. Salt Diffusion into the Claw Meat

The salt content of the claw meat was determined to assess the ability of HPP to promote the diffusion of small solutes (e.g., sodium chloride) (Table 4).

Both HPP and cooking increased the salt content of claw meat significantly (*p* < 0.05) when a 5% (*w*/*v*) sea salt brine was used as the pressure-transmitting or cooking medium. Moreover, the pressure treatments HPP300/4 and HPP500/2 resulted in significantly (*p* < 0.05) higher salt content compared to their counterparts cooked in the brine at the same concentration (5% *w*/*v* sea salt). The salt content increased with treatment pressure and time, up to 50% in the meat of HPP500/2 claws compared with the meat of raw (i.e., untreated) claws.

Previous studies have demonstrated that HPP could be used as a processing aid in enhancing salt diffusion in meat products (e.g., turkey or chicken) infused in brine before the high-pressure treatment [94,95,96]. Nonetheless, to the best of our knowledge, the present study was notably the first one in which the effect of HPP on salt diffusion was tested on crustacean species and with the product directly immersed in the salt solution (i.e., the pressure-transmitting fluid containing 5% (*w*/*v*) sea salt) during the high-pressure treatment. It should also be highlighted that, differently from HPP, ultrasound-assisted cooking in water with 5% (*w*/*v*) sea salt was not effective in increasing the diffusion of sea salt into the meat of the body or of the claws of edible crab [97].

The results of the present study indicate that HPP enhances the diffusion of small solutes through the pore canals of the exoskeleton of crabs. In this way, high-pressure treatments may be exploited in crabs, or more in general, crustacean species, to allow for the penetration into the meat of functional compounds, such as anti-microbial agents [32], inhibitors of enzymatic discoloration (e.g., melanosis) [98], or water binders to increase the yield [32].

## 4. Conclusions

High-pressure processing (HPP) was an effective non-thermal processing method alternative to cooking for achieving protein denaturation and, therefore, the detachment of the claw muscle from the exoskeleton. HPP promoted the ingress of water into the intra-myofibrillar space of claw muscle, leading to higher processing yield than cooking and after the application of thermal pasteurization.

All the HPP treatments (i.e., 300 MPa/2 min, 300 MPa/4 min, and 500 MPa/2 min) resulted in meat with lower levels of total volatile basic nitrogen as compared with thermally processed samples. The nutritional value of the meat in terms of its fatty acid composition was not affected by HPP, while higher pressure and longer time led, instead, to increasing microbial inactivation. The best visual quality appearance was obtained for the meat of claws treated at 300 MPa for 4 min, which showed no discolorations, and after a subsequent thermal pasteurization, high color similarity with the conventionally processed product (i.e., cooked and cooked-pasteurized claw meat).

Conducting the pressure treatments on the chelipeds immersed in a pressure-transmitting fluid constituted of water with 5% (*w*/*v*) sea salt allowed for a significant (*p* < 0.05) increase in the salt concentration of the claw meat. This suggests that HPP may be used to facilitate the inclusion of small solutes with specific technological functions (e.g., anti-melanosis, water binding, or anti-microbial compounds) into the product.

The application of HPP at 300 MPa for 4 min showed its potential as a possible pre-treatment for producing added-value claw meat from edible crab in the form of uncooked ready-to-heat or pasteurized ready-to-eat meat products. 

Nonetheless, although the present study offered a comprehensive overview of the impact of HPP on the physical, chemical, microbial, and nutritional quality aspects of crab meat, further research is required to investigate more closely the microbial stability and to evaluate the sensory properties and consumer acceptability of such products before they can be brought to market. Future work should also include evaluating the quality parameters of the meat of HPP-treated crab claws during chilled or frozen storage.

As the present study is based on high-pressure treatments conducted in an industrial large-scale HPP unit, these results may be particularly useful to crab processors for implementations at the commercial level.

## Figures and Tables

**Table 1:** Processing yield, moisture, protein, and ash content, pH, total volatile basic nitrogen (TVB-N), and color parameters (L*, a*, b*, ΔE*_cooked_, ΔE*_cooked+P_) of the meat extracted from raw (i.e., untreated) and processed edible crab claws.

	Raw	Cooked	HPP300/2	HPP300/4	HPP500/2	Cooked+P	HPP300/2+P	HPP300/4+P	HPP500/2+P
Processing yield (%)	0.0	−5.2 ± 1.2 ^c^	0.9 ± 0.6 ^a^	1.3 ± 0.9 ^a^	1.2 ± 1.0 ^a^	−7.7 ± 1.0 ^d^	−1.2 ± 0.4 ^b^	−0.6 ± 0.4 ^b^	−1.4 ± 0.9 ^b^
Moisture (%)	76.9 ± 1.6 ^bcd^	74.7 ± 1.2 ^de^	78.5 ± 2.1 ^bc^	79.3 ± 2.0 ^ab^	81.8 ± 1.8 ^a^	72.4 ± 1.1 ^e^	76.2 ± 0.9 ^cd^	78.6 ± 1.6 ^bc^	76.5 ± 1.9 ^cd^
Protein (%)	18.0 ± 0.7 ^bc^	18.6 ± 0.6 ^ab^	16.7 ± 0.6 ^cd^	16.5 ± 0.7 ^cd^	15.2 ± 0.7 ^d^	19.9 ± 1.0 ^a^	17.9 ± 0.8 ^bc^	18.3 ± 0.8 ^b^	16.2 ± 1.2 ^d^
Ash (%)	2.26 ± 0.08 ^cd^	2.64 ± 0.10 ^ab^	2.05 ± 0.09 ^de^	2.06 ± 0.06 ^d^	1.83 ± 0.18 ^e^	2.74 ± 0.17 ^a^	2.55 ± 0.10 ^ab^	2.51 ± 0.09 ^b^	2.48 ± 0.17 ^bc^
pH	6.92 ± 0.04 ^f^	7.36 ± 0.04 ^b^	7.01 ± 0.03 ^e^	7.04 ± 0.02 ^de^	7.09 ± 0.02 ^d^	7.46 ± 0.03 ^a^	7.27 ± 0.03 ^c^	7.30 ± 0.02 ^c^	7.41 ± 0.03 ^ab^
TVB-N (mg N/100 g)	19.3 ± 1.7 ^e^	32.1 ± 1.9 ^d^	18.6 ± 0.5 ^e^	17.7 ± 1.1 ^e^	16.3 ± 0.7 ^e^	52.9 ± 2.9 ^a^	35.1 ± 2.2 ^cd^	36.8 ± 2.8 ^c^	40.7 ± 2.1 ^b^
*Color*									
L*	n.d.	76.93 ± 0.48 ^d^	66.89 ± 2.03 ^f^	69.62 ± 0.62 ^e^	77.78 ± 0.59 ^bcd^	77.36 ± 0.29 ^cd^	77.97 ± 0.42 ^bc^	78.42 ± 0.91 ^b^	82.50 ± 0.91 ^a^
a*	n.d.	−3.55 ± 0.08 ^c^	−1.30 ± 0.19 ^a^	−1.03 ± 0.18 ^a^	−2.82 ± 0.56 ^b^	−2.78 ± 0.07 ^b^	−2.78 ± 0.22 ^b^	−2.74 ± 0.41 ^b^	−2.71 ± 0.19 ^b^
b*	n.d.	6.07 ± 0.37 ^b^	0.40 ± 1.07 ^c^	−0.48 ± 0.64 ^d^	−2.01 ± 0.91 ^e^	6.52 ± 0.20 ^b^	7.48 ± 0.95 ^a^	6.86 ± 1.11 ^ab^	6.77 ± 1.09 ^ab^
ΔE*_cooked_	n.d.	0.0	11.8 ± 1.8 ^a^	10.1 ± 0.4 ^ab^	8.2 ± 0.8 ^bc^	1.0 ± 0.2 ^d^	2.0 ± 0.5 ^d^	1.9 ± 0.6 ^d^	5.7 ± 0.6 ^c^
ΔE*_cooked+P_	n.d.	1.0 ± 0.1 ^d^	12.2 ± 1.8 ^a^	10.6 ± 0.4 ^ab^	8.6 ± 0.8 ^b^	0.0	1.2 ± 0.6 ^d^	1.2 ± 0.6 ^d^	5.2 ± 0.6 ^c^

Note. Results are expressed as mean values (±standard deviation); n.d. = not determined. Different superscript letters within the same row indicate significantly different (*p* < 0.05) mean values. Sample codes: HPP300/2 and HPP300/4, high-pressure processed at 300 MPa for 2 and 4 min, respectively; HPP500/2, high-pressure processed at 500 MPa for 2 min; +P, thermal pasteurization after cooking or high-pressure processing.

**Table 2:** Peak center (ms) and area proportion (%) of the water relaxation components from continuous distribution curves of transverse relaxation time (*T*_2_) obtained by LF-NMR analysis of meat extracted from raw (i.e., untreated) and processed edible crab claws.

	*T*_2b_		*T*_21_		*T*_22_		*T*_22′_	
	Center (ms)	Area (%)	Center (ms)	Area (%)	Center (ms)	Area (%)	Center (ms)	Area (%)
Raw	2.5 ± 0.4 ^c^	0.1 ± 0.0	37.8 ± 1.9 ^cd^	37.4 ± 8.0 ^bc^	195.8 ± 20.2 ^de^	45.9 ± 10.2 ^a^	1004.3 ± 132.2 ^a^	16.7 ± 3.1 ^b^
Cooked	5.8 ± 2.2 ^ab^	0.1 ± 0.0	42.5 ± 1.4 ^bc^	37.1 ± 6.2 ^bc^	290.8 ± 17.7 ^c^	21.4 ± 4.8 ^bcd^	548.4 ± 97.1 ^c^	42.0 ± 10.2 ^a^
HPP300/2	4.0 ± 0.9 ^bc^	0.1 ± 0.0	46.1 ± 6.1 ^bc^	79.8 ± 4.8 ^a^	483.0 ± 61.3 ^b^	11.7 ± 1.3 ^de^	863.7 ± 96.8 ^ab^	14.2 ± 2.2 ^b^
HPP300/4	4.1 ± 0.9 ^bc^	0.1 ± 0.1	45.3 ± 11.3 ^bc^	81.1 ± 4.8 ^a^	596.6 ± 102.2 ^a^	18.8 ± 4.8 ^cde^		
HPP500/2	7.5 ± 1.6 ^a^	0.2 ± 0.0	72.3 ± 7.8 ^a^	90.4 ± 1.9 ^a^	589.0 ± 21.3 ^a^	9.4 ± 0.9 ^e^		
Cooked+P	2.8 ± 0.3 ^c^	0.1 ± 0.0	35.2 ± 1.2 ^d^	39.4 ± 6.4 ^b^	176.7 ± 18.7 ^e^	29.9 ± 3.7 ^b^	326.1 ± 54.2 ^d^	30.6 ± 4.4 ^ab^
HPP300/2+P	3.8 ± 0.7 ^bc^	0.1 ± 0.0	45.4 ± 1.3 ^bc^	29.9 ± 11.1 ^bc^	232.2 ± 7.8 ^cde^	23.1 ± 7.3 ^bc^	800.2 ± 78.9 ^b^	47.0 ± 17.0 ^a^
HPP300/4+P	4.5 ± 0.7 ^bc^	0.1 ± 0.0	48.3 ± 2.0 ^b^	37.0 ± 8.1 ^bc^	265.5 ± 14.2 ^cd^	22.4 ± 4.9 ^bc^	816.3 ± 146.0 ^ab^	40.6 ± 11.7 ^a^
HPP500/2+P	3.9 ± 0.6 ^bc^	0.1 ± 0.0	47.2 ± 4.0 ^bc^	26.1 ± 4.6 ^c^	249.9 ± 13.7 ^cde^	31.4 ± 4.6 ^b^	826.7 ± 41.0 ^b^	42.4 ± 7.2 ^a^

Note. Results are expressed as mean values (±standard deviation). Different superscript letters within the same column indicate significantly different (*p* < 0.05) mean values. Sample codes: HPP300/2 and HPP300/4, high-pressure processed at 300 MPa for 2 and 4 min, respectively; HPP500/2, high-pressure processed at 500 MPa for 2 min; +P, thermal pasteurization after cooking or high-pressure processing.

**Table 3:** Fatty acid composition (percentage of the total detected fatty acids) of meat extracted from raw (i.e., untreated) and processed edible crab claws.

Fatty Acid	Raw	Cooked	HPP300/2	HPP300/4	HPP500/2	Cooked+P	HPP300/2+P	HPP300/4+P	HPP500/2+P
16:0	12.0 ± 0.9 ^ab^	12.2 ± 0.8 ^ab^	11.3 ± 0.7 ^b^	12.4 ± 0.3 ^ab^	12.1 ± 0.8 ^ab^	12.7 ± 0.5 ^a^	11.6 ± 0.6 ^ab^	12.4 ± 0.2 ^ab^	12.2 ± 0.9 ^ab^
18:0	4.3 ± 0.5 ^ab^	5.4 ± 1.0 ^a^	4.6 ± 0.2 ^ab^	4.1 ± 0.4 ^b^	4.3 ± 0.5 ^ab^	4.9 ± 1.2 ^ab^	4.7 ± 0.2 ^ab^	4.2 ± 0.4 ^b^	4.4 ± 0.4 ^ab^
∑ SFA ^1^	18.3 ± 0.7	19.2 ± 1.4	18.0 ± 0.8	18.2 ± 0.8	19.0 ± 2.6	19.2 ± 0.7	18.3 ± 1.1	18.3 ± 0.7	18.2 ± 1.0
16:1	9.1 ± 0.9	7.2 ± 2.3	6.7 ± 2.3	7.3 ± 1.1	6.8 ± 1.9	8.2 ± 2.6	6.0 ± 1.5	7.2 ± 1.2	6.8 ± 2.1
18:1*n*−9*c*	18.5 ± 2.7	21.2 ± 1.1	18.6 ± 3.7	18.9 ± 0.6	19.7 ± 2.0	20.5 ± 0.9	17.9 ± 3.8	18.8 ± 0.8	19.6 ± 1.2
18:1*n*−7	7.1 ± 1.1	5.5 ± 0.2	6.7 ± 1.0	6.6 ± 0.2	6.6 ± 0.9	6.4 ± 1.3	6.6 ± 1.0	6.5 ± 0.2	6.6 ± 1.1
∑ MUFA ^2^	36.7 ± 1.7 ^a^	35.5 ± 2.0 ^ab^	34.6 ± 0.1 ^ab^	34.7 ± 1.6 ^ab^	34.8 ± 1.9 ^ab^	36.6 ± 3.0 ^a^	33.0 ± 1.1 ^b^	34.5 ± 1.9 ^ab^	34.7 ± 2.4 ^ab^
20:4*n*−6	4.5 ± 0.7	5.3 ± 1.0	6.2 ± 1.8	6.1 ± 0.6	5.8 ± 1.9	4.4 ± 0.4	5.9 ± 1.7	5.9 ± 0.5	5.8 ± 1.6
∑ (*n*−6)	6.0 ± 0.8	8.5 ± 0.6	8.7 ± 2.3	8.2 ± 1.0	7.9 ± 3.0	6.5 ± 1.8	8.4 ± 2.0	8.0 ± 0.9	7.9 ± 2.6
20:5*n*−3 (EPA)	27.4 ± 2.1	24.3 ± 2.2	25.7 ± 1.8	25.2 ± 2.3	26.9 ± 1.0	26.3 ± 2.9	26.9 ± 3.0	25.7 ± 1.7	27.5 ± 1.6
22:5*n*−3	1.6 ± 0.9	0.7 ± 0.4	2.1 ± 1.4	1.7 ± 0.5	0.9 ± 0.2	0.8 ± 0.2	2.0 ± 1.1	1.5 ± 0.4	1.0 ± 0.3
22:6*n*−3 (DHA)	10.0 ± 1.4	11.8 ± 0.6	10.8 ± 1.2	12.0 ± 2.0	10.5 ± 2.5	10.6 ± 1.8	11.4 ± 1.9	11.9 ± 1.8	10.7 ± 2.1
∑ (*n*−3)	39.0 ± 1.6	36.9 ± 1.5	38.6 ± 1.9	38.9 ± 0.8	38.3 ± 2.0	37.7 ± 1.4	40.3 ± 2.3	39.1 ± 0.6	39.2 ± 0.8
∑ PUFA ^3^	45.0 ± 1.7 ^ab^	45.3 ± 1.6 ^ab^	47.4 ± 0.7 ^ab^	47.1 ± 1.6 ^ab^	46.2 ± 4.1 ^ab^	44.2 ± 2.4 ^b^	48.7 ± 0.6 ^a^	47.1 ± 1.5 ^ab^	47.1 ± 3.4 ^ab^
EPA + DHA (mg/g)	0.92 ± 0.07 ^d^	1.07 ± 0.10 ^cd^	1.08 ± 0.17 ^bcd^	1.31 ± 0.22 ^abc^	0.94 ± 0.12 ^d^	1.07 ± 0.07 ^cd^	1.36 ± 0.21 ^ab^	1.41 ± 0.20 ^a^	1.13 ± 0.11 ^abcd^
∑ (*n*−3)/∑ (*n*−6)	6.6 ± 1.0	4.4 ± 0.4	4.7 ± 1.6	4.8 ± 0.6	5.5 ± 2.3	6.2 ± 1.7	5.0 ± 1.6	4.9 ± 0.5	5.4 ± 2.0
∑ PUFA ^3^/∑ SFA^1^	2.5 ± 0.1	2.4 ± 0.2	2.6 ± 0.2	2.6 ± 0.2	2.5 ± 0.5	2.3 ± 0.1	2.7 ± 0.2	2.6 ± 0.1	2.6 ± 0.3
PI	3.1 ± 0.3	3.0 ± 0.4	3.2 ± 0.2	3.0 ± 0.2	3.1 ± 0.2	2.9 ± 0.2	3.3 ± 0.2	3.0 ± 0.1	3.2 ± 0.3

Note. Results are expressed as mean values (±standard deviation). Different superscript letters within the same row indicate significantly different (*p* < 0.05) mean values. Sample codes: HPP300/2 and HPP300/4, high-pressure processed at 300 MPa for 2 and 4 min, respectively; HPP500/2, high-pressure processed at 500 MPa for 2 min; +P, thermal pasteurization after cooking or high-pressure processing. Abbreviations: DHA, docosahexaenoic acid; EPA, eicosapentaenoic acid; MUFA, monounsaturated fatty acid; PI, polyene index, calculated as ((20:5*n*−3 + 22:6*n*−3)/(16:0)); PUFA, polyunsaturated fatty acid; SFA, saturated fatty acid. ^1^ It also includes the SFAs accounting for less than 2% of the total detected fatty acids (i.e., 14:0, 15:0, and 17:0). SFAs below 0.2% (i.e., 12:0, 20:0, 21:0, and 22:0) were not considered. ^2^ It also includes the MUFAs accounting for less than 2% of the total detected fatty acids (i.e., 17:1, 18:1*n*−9*t*, and 20:1*n*−9). MUFAs below 0.2% (i.e., 15:1 and 22:1*n*−9) were not considered. ^3^ It also includes the PUFAs accounting for less than 2% of the total detected fatty acids (i.e., 18:2*n*−6 and 20:2*n*−6). PUFAs below 0.2% (i.e., 18:3*n*−6, 18:3*n*−3, 20:3*n*−6, 20:3*n*−3, and 22:2*n*−6) were not considered.

**Table 4:** Salt content (%) in the meat extracted from raw (i.e., untreated) claws and from claws cooked or high-pressure processed in a medium constituted of fresh water added of sea salt at a concentration of 1 or 5% (*w*/*v*).

	Sea Salt Concentration (*w*/*v*) in the Processing Medium	
	1%	5%
Raw	1.10 ± 0.09 *	1.10 ± 0.09 *
Cooked	1.08 ± 0.07 ^ef^*	1.22 ± 0.06 ^cd^
HPP300/2	1.02 ± 0.07 ^f^*	1.31 ± 0.08 ^c^
HPP300/4	1.08 ± 0.07 ^ef^*	1.49 ± 0.07 ^b^
HPP500/2	1.15 ± 0.06 ^de^*	1.71 ± 0.10 ^a^

Note. Results are expressed as mean values (±standard deviation). Different superscript letters indicate significantly different (*p* < 0.05) mean values. The symbol (*) indicates the mean values which are not significantly different (*p* ≥ 0.05) from the mean value observed for the meat from raw claws. Sample codes: HPP300/2 and HPP300/4, high-pressure processed at 300 MPa for 2 and 4 min, respectively; HPP500/2, high-pressure processed at 500 MPa for 2 min.

## Data Availability

The data presented in this study are available on reasonable request from the corresponding author.
